# Supplementary material for: MetaQTL: a package of new computational methods for the meta-analysis of QTL mapping experiments
Source: BMC Bioinformatics. 2007 Feb 8;8:49. doi: 10.1186/1471-2105-8-49 (PMC1808479; doi:10.1186/1471-2105-8-49)
Supplement: Additional File 7 — MetaQTL Package : jar file and tutorial. This Zip archive contains both the MetaQTL JAR file and the files of the tutorial. [file 1471-2105-8-49-S7.zip › org.inra.metaqtl/doc/org/metaqtl/util/LocusProperties.html]

LocusProperties


|  |  |  |
| --- | --- | --- |
| **Overview** \| **Package** \| **Class** \| **Use** \| **Tree** \| **Deprecated** \| **Index** \| **Help** | |  |
| **PREV CLASS**   **NEXT CLASS** | **FRAMES**    **NO FRAMES**     **All Classes** |  |
| SUMMARY: NESTED \| FIELD \| CONSTR \| METHOD | DETAIL: FIELD \| CONSTR \| METHOD |  |


---


## org.metaqtl.util Class LocusProperties

```
java.lang.Object
  org.metaqtl.util.LocusProperties
```

---

``` public final class LocusProperties extends java.lang.Object ```

**Author:**
:   jveyrier
    TODO To change the template for this generated type comment go to
    Window - Preferences - Java - Code Style - Code Templates

---

**Constructor Summary**

`LocusProperties()`


| **Method Summary** | |
| --- | --- |
| `static int` | `getMetaOccurence(IBioLocus locus)` |
| `static boolean` | `isDubiousOrderLocus(IBioLocus locus)`             Returns true if the given locus has a property order.dubious set to one, false otherwise |

**Methods inherited from class java.lang.Object**

`clone, equals, finalize, getClass, hashCode, notify, notifyAll, toString, wait, wait, wait`

**Constructor Detail**

### LocusProperties

```
public LocusProperties()
```


**Method Detail**

### isDubiousOrderLocus

```
public static boolean isDubiousOrderLocus(IBioLocus locus)
```

:   Returns true if the given locus has a property order.dubious
    set to one, false otherwise

    :   **Parameters:**: `map` - **Returns:**

---


### getMetaOccurence

```
public static int getMetaOccurence(IBioLocus locus)
```


---


|  |  |  |
| --- | --- | --- |
| **Overview** \| **Package** \| **Class** \| **Use** \| **Tree** \| **Deprecated** \| **Index** \| **Help** | |  |
| **PREV CLASS**   **NEXT CLASS** | **FRAMES**    **NO FRAMES**     **All Classes** |  |
| SUMMARY: NESTED \| FIELD \| CONSTR \| METHOD | DETAIL: FIELD \| CONSTR \| METHOD |  |


---
